# Outcomes in patients with dual antegrade conduction in the atrioventricular node: insights from a multicentre observational study

**DOI:** 10.1007/s00392-020-01596-y

**Published:** 2020-01-30

**Authors:** Jens Hartmann, Christiane Jungen, Sebastian Stec, Niklas Klatt, Stephan Willems, Hisaki Makimoto, Daniel Steven, Helmut Pürerfellner, Martin Martinek, Christian Meyer

**Affiliations:** 1 Department of Cardiology, Asklepios Klinik St. Georg, Hamburg, Germany; 2 Department of Cardiology-Electrophysiology, University Heart and Vascular Center, Hamburg, Germany; 3 Subcarpathian Center for Cardiovascular Intervention, G.V.M. Carint, Sanok, Poland; 4 Medinice Research and Development Centre, Aeropolis-Jasionka, Rzeszow, Poland; 5 ELMedica EP-Network, Kielce, Poland; 6 Department of Cardiology, Pulmonology and Vascular Medicine, Medical Faculty, Heinrich Heine University, Düsseldorf, Germany; 7 Department of Cardiology-Electrophysiology, University Hospital Cologne, Cologne, Germany; 8 Department of Cardiology, Academic Teaching Hospital, Ordensklinikum Linz Elisabethinen, Linz, Austria; 9 DZHK (German Centre for Cardiovascular Research), Partner Site Hamburg/Kiel/Lübeck, Berlin, Germany

**Keywords:** AVNRT, Ablation, Atrial fibrillation, DAVNNT, Double fire, Slow pathway

## Abstract

**Background:**

Supraventricular tachycardias induced by dual antegrade conduction via the atrioventricular (AV) node are rare but often misdiagnosed with severe consequences for the affected patients. As long-term follow-up in these patients was not available so far, this study investigates outcomes in patients with dual antegrade conduction in the AV node.

**Methods and results:**

In this multicentre observational study, patients from six European centres were studied. Catheter ablation was performed in 17 patients (52 ± 16 years) with dual antegrade conduction via both AV nodal pathways between 2012 and 2018. Patients with the final diagnosis of a manifest dual AV nodal non-re-entrant tachycardia had a mean delay of the correct diagnosis of over 1 year (range 2–31 months). Two patients received prescription of non-indicated oral anticoagulation, two further patients suffered from inappropriate shocks of an implantable cardioverter defibrillator. In 12 patients, a co-existence of dual antegrade and re-entry conduction in the AV node was present. Mean fast pathway conduction time was 138 ± 61 ms and mean slow pathway conduction time was 593 ± 134 ms. Successful radiofrequency catheter ablation was performed in all patients. Post-procedurally oral anticoagulation was discontinued, without detection of cerebrovascular events or atrial fibrillation during a long-term follow-up of median 17 months (range 6–72 months).

**Conclusion:**

This first multicentre study investigating patients with supraventricular tachycardia and dual antegrade conduction in the AV node demonstrates that catheter ablation is safe and effective while long-term patient outcome is good. Autonomic tone dependent changes in ante- vs. retrograde conduction via slow and/or fast pathway can challenge the diagnosis and therapy in some patients.

**Graphic abstract:**

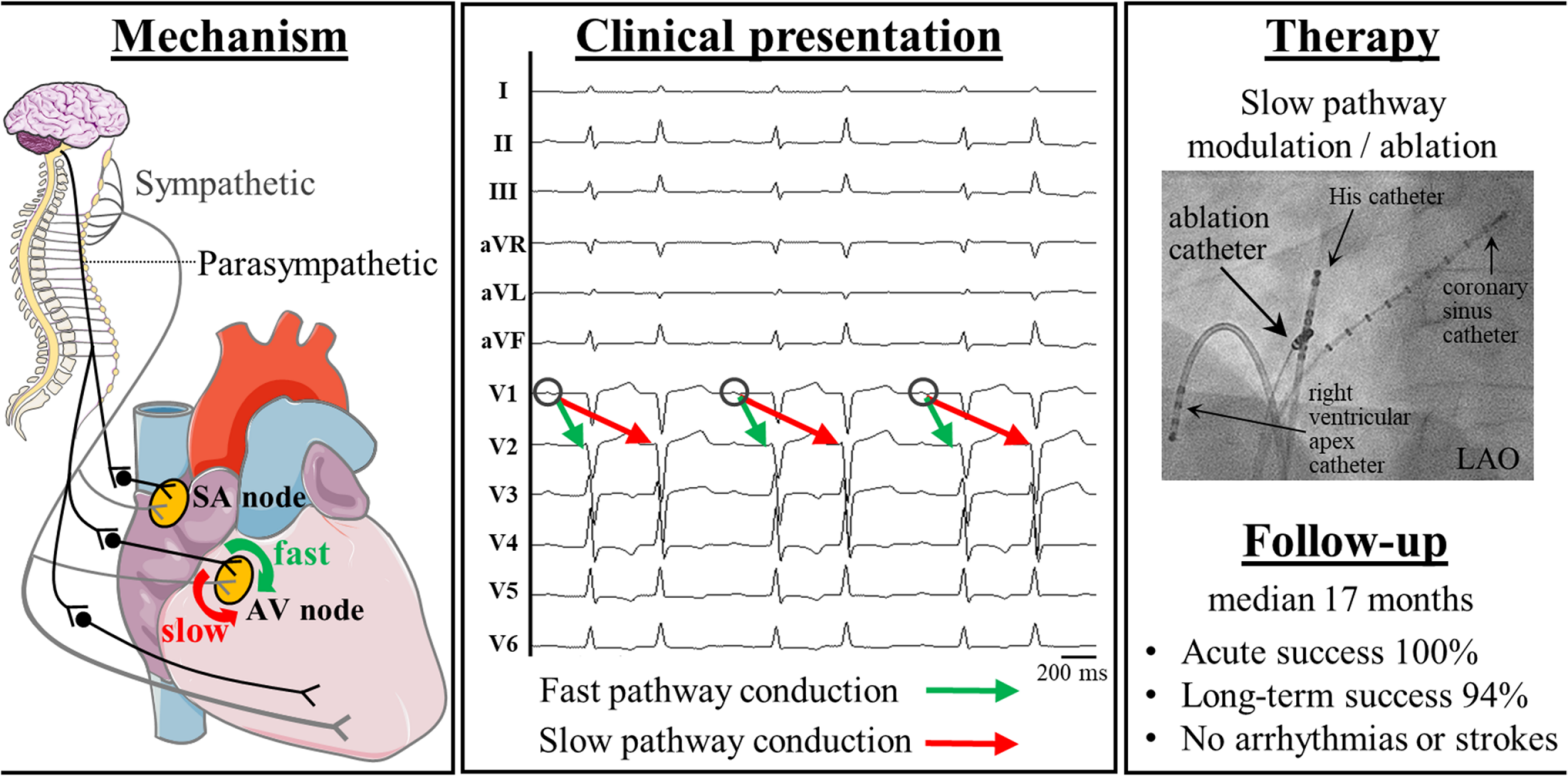

## Introduction

**Fig. 1:**
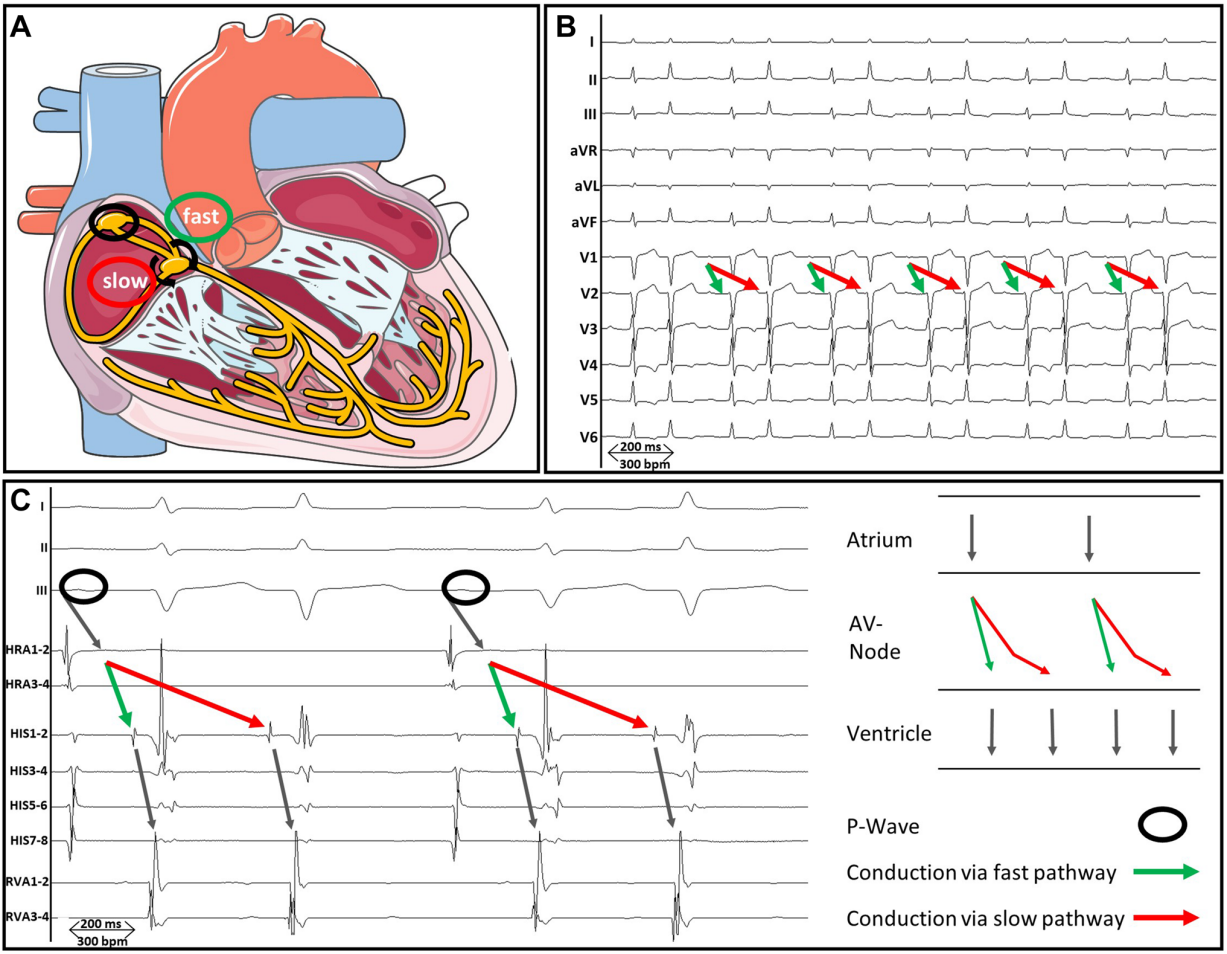
Schematic and anatomical depiction of a dual antegrade conduction in the AV node. **a** The schematic drawing of the conduction system (yellow) of the heart depicts the dual AV nodal physiology, which is a prerequisite for dual antegrade conduction in the AV node. The green marker highlights conduction via the fast pathway, the red marker illustrates conduction via the slow pathway. **b** A 12-lead electrocardiogram of a dual AV nodal non-re-entrant tachycardia (DAVNNT) is presented. Note the single P wave followed by two QRS complexes. **c** An exemplary tracing of intracardiac electrograms during an electrophysiological study of a patient with DAVNNT is depicted. Catheters have been placed at the high right atrium (HRA), the His bundle (HIS) and the right ventricular apex (RVA). One atrial signal is followed by two ventricular responses as seen in the HRA, HIS and RVA catheters

The atrioventricular (AV) nodal area can consist of two areas that have different conduction characteristics, namely the fast and the slow pathway. This dual physiology is the basis for AV nodal re-entrant tachycardias (AVNRTs), which are the predominant form of supraventricular tachycardias comprising about 56% of affected patients [1–3]. Catheter ablation of AVNRT is proposed as first-line therapy with high success (> 90%) and relatively low complication rates (< 5%) [2, 4–6]. A functional dissociation of the AV node is the prerequisite for an AVNRT and is present in up to 35% of the general population [7]. Besides the AVNRT re-entry mechanism, the rarely diagnosed entity of dual antegrade conduction via both pathways is now becoming well known in consequence of numerous case reports and as it was just recently mentioned for the first time in the guidelines of the European Society of Cardiology [8–11]. This dual AV nodal non-re-entrant tachycardia (DAVNNT), which is also known as double ventricular response, one to two tachycardia or simply ‘double fire’ has been first reported in 1975 [8, 9]. Since then, besides case reports only one small single-centre experience including five patients [10] (all together less than 80 patients) [8, 9, 12, 13] have been described. The suspected diagnosis can be made by a 12-lead electrocardiogram (ECG), in which a P wave is followed by two interpolated QRS complexes (Fig. 1) [8, 9]. This diagnostic tool has been proposed as gold standard [8], but the correct diagnosis may be complicated by its heterogeneous appearance leading to several differential diagnosis consisting of atrial fibrillation (AF), sporadic junctional extrasystoles, parasystoles, ventricular tachycardia or AVNRT with retrograde 1:2 conduction block [8, 9]. The rarity and possible mimicking of other, more common arrhythmias has led to initial misdiagnoses in about 70% of all published cases [8] and to an underestimation of this ‘chameleon of the AV node’ [8–10, 14]. Furthermore, patients treated for misdiagnosed AF by oral anticoagulation or non-indicated implantations of implantable cardioverter defibrillators (ICDs)—even with the appearance of inadequate shocks—emphasize the relevance of a better understanding and implementation of this phenomenon in daily clinical routine [8–10]. Catheter ablation of the slow pathway seems to be acutely as effective and safe as in patients with AVNRT, but data on long-term patient outcome are not reported [8–10]. Therefore, we report the first multicentre study investigating patients with supraventricular tachycardia and dual antegrade conduction in the AV node.

## Methods

### Study design and patient population

In this international multicentre observational study, we investigated all subsequent patients with dual antegrade conduction in the AV node resulting in DAVNNT from six European centres between January 2012 and August 2018. Patient data processing was conducted with approval from the local ethics committee and in accordance with current legislation.

Diagnosis of dual antegrade conduction was obtained by (1) clinical symptoms comprising of palpitations and/or tachycardia, (2) a 12-lead ECG and/or Holter-ECG consistent with dual antegrade conduction in the AV node, and (3) verification of dual antegrade conduction in the AV node during an electrophysiological study [8–10]. Electrophysiological studies and patient data were assessed via a standardised questionnaire. The initial (mis-)diagnosed arrhythmia, the time to correct diagnosis, inappropriate therapies as well as medical treatment were assessed besides patient history and characteristics.

Since tachycardia-induced cardiomyopathy, defined as a newly reduced left ventricular ejection fraction (LVEF) without other cardiac abnormalities that normalized after ablation (LVEF < 50% with improvement to > 50% or LVEF 50–55% with improvement > 10%), has been reported in patients with dual antegrade conduction, the data were also investigated for patients with this suspected diagnosis [8, 15, 16].

### Electrophysiological study and ablation

The electrophysiological studies were performed in accordance with the current guidelines following established standards [2, 17, 18]. The time intervals during dual antegrade conduction, especially the intervals between the beginning of the atrial activation and the corresponding activation of the His–Purkinje system, resembling the atrial activation and fast pathway conduction time (AH_1_) and the atrial activation and slow pathway conduction time (AH_2_) were assessed. The typical conduction pattern of a dual ventricular response to one atrial activation (as seen as an AH_1_V_1_H_2_V_2_-sequence in the intracardial electrograms during an electrophysiological study; atrial signal (A), HIS-signal (H), ventricular signal (V)), and also a dual ventricular response to the first atrial activation followed by an AV nodal echo beat (A_1_H_1_V_1_H_2_V_2_A_2_-sequence in the intracardial electrograms) were defined as verification of the diagnosis of dual antegrade conduction in the AV node [8–10]. After verification of diagnosis, patients were treated with catheter-based ablation via application of radiofrequency impulses to the right atrial posteroseptal area, attempting to achieve slow pathway modulation or ablation [8, 19, 20]. Ablation has been performed with 4-mm tip catheters (Biotronik AlCath Blue TC G Full Circle; Osypka Cerablate Easy Classic Curve, 60 mm; Biosense Webster ThermoCool surround flow D-curve). In some cases, a 63-cm sheath was additionally used to facilitate reachability and stability of the slow pathway region, while an F curve catheter may be another useful alternative for optimal and stable catheter positioning. Ablation energy ranged from 20 to 40 W in all but one case, in which a maximum energy of 50 W has been applied. During the ablation in the slow pathway region typical occurrence of junctional beats or an accelerated junctional rhythm could be observed. Whereas ablation of the slow pathway was defined as elimination of slow pathway conduction, modulation was assumed as an ablation until conduction differences in the slow pathway occur with persistent dual AV nodal physiology, defined by the presence of an AH jump (> 50 ms) or a single AV nodal echo beat [19, 20]. Acute procedure-related major adverse events were defined as death of any cause, post-procedure haemorrhage requiring blood transfusion, sepsis, aspiration, cardiac surgery, stroke, pulmonary embolism, cardiogenic shock, pericardial effusion, indication for pacemaker implantation, or major groin complications requiring surgical intervention. Minor adverse events were defined as post-procedure haemorrhage not requiring transfusion, post-procedural transient AV block without indication for pacemaker implantation, and minor groin complications not requiring vascular intervention.

### Follow-up

All patients were assessed with a 12-lead ECG and/or 24-h Holter monitoring during a follow-up outpatient visit. Besides clinical symptoms, recurrences of supraventricular tachycardias and the occurrence of cerebrovascular events were assessed [21].

### Statistical analyses

Statistical analysis was performed using Microsoft Excel and GraphPad Prism 6.0 (GraphPad Inc., La Jolla, California, USA). Continuous variables are reported as mean and standard deviation (SD) or median and range. Categorical variables are presented as counts or percentages.

## Results

### Patient characteristics

Patient characteristics are presented in Table 1. Seventeen patients (ten male) were diagnosed with dual antegrade conduction in the AV node. Patients’ age ranged from ten to 80 years with a mean age of 52 ± 16 years with mean LVEF of 52 ± 12%. In five patients, a coronary artery disease was present.

**Table 1:** Baseline patient characteristics

Patients (*n*)	17
Sex (male), *n* (%)	10 (59)
Age (years), mean ± SD	52 ± 16
Left ventricular ejection fraction (%), mean ± SD	52 ± 12
Coronary artery disease, *n* (%)	5 (29)
Hyperlipidemia, *n* (%)	6 (35)
Diabetes mellitus, *n* (%)	1 (6)
Stroke/transitory ischemic attack, *n* (%)	2 (12)
Hypertension, *n* (%)	6 (35)
Chronic obstructive pulmonary disease	0
Sleep apnea	0
History of smoking, *n* (%)	3 (18)

### Diagnosis, misdiagnosis and electrophysiological study

**Fig. 2:**
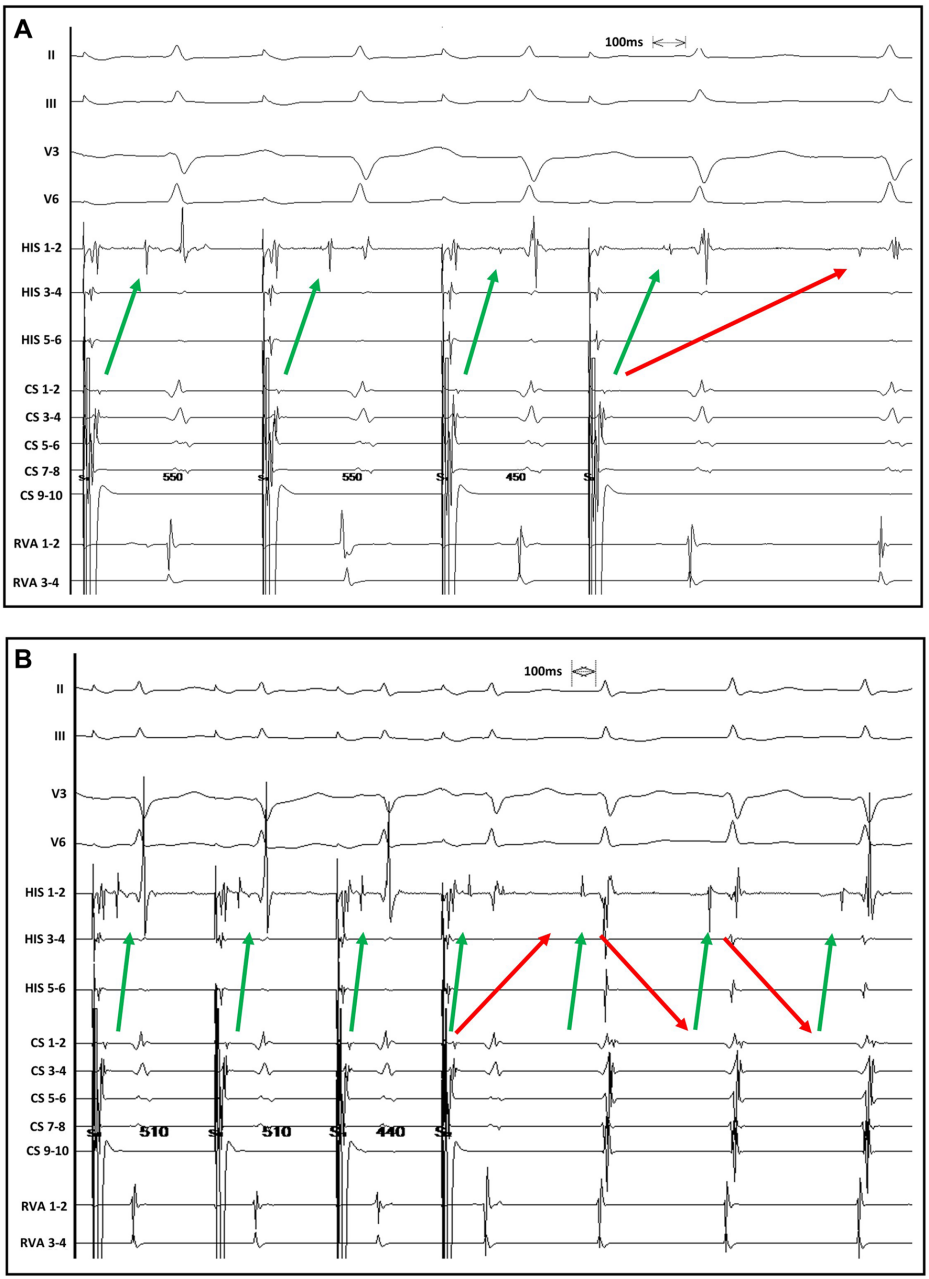
Induction of double ventricular response and AVNRT in the same patient. Intracardiac electrograms with catheters placed at the His bundle (HIS), the coronary sinus (CS) and the right ventricular apex (RVA) are presented. The green marker highlights conduction via the fast pathway, the red marker illustrates conduction via the slow pathway. Under baseline conditions, VA-dissociation, a functional dissociation of conduction within the AV node and spontaneous dual antegrade conduction in the AV node could be demonstrated. After administration of orciprenaline VA-conduction was present with a retrograde Wenckebach cycle length of 320 ms. Subsequently, double ventricular response and a typical AVNRT were inducible by programmed atrial stimulation. Diagnosis of typical AVNRT was confirmed by ventricular overdrive pacing with an AH response, short septal VA interval and negative preceding manoeuvre. **a** At baseline conditions programmed atrial stimulation via a proximal CS electrode (S1: 550 ms, S2: 450 ms) results in a double ventricular response with an A_1_H_1_V_1_H_2_V_2_ sequence. **b** After administration of orciprenaline programmed atrial stimulation (S1: 510 ms, S2: 440 ms) induces an AVNRT with an A_1_H_1_V_1_H_2_V_2_A_2_-sequence

Twelve of the 17 patients showed a typical 12-lead ECG of continuous dual antegrade conduction with P waves followed by two QRS complexes on admission. In the remaining five patients with suspected supraventricular tachycardia, the dual antegrade conduction was diagnosed during an electrophysiological study with observation of 1:2 conduction following programmed stimulation, induction or ablation of an AVNRT with an A_1_H_1_V_1_H_2_V_2_A_2_ sequence (Fig. 2). The arrhythmia was sustained in at least two of the patients. Nine out of twelve patients (75%) with a typical DAVNNT in the 12-lead ECG were primarily misdiagnosed. The average time between initial symptoms and correct diagnosis was 13 ± 11 months (range 2–31 months). The misdiagnoses consisted of AF (*n* = 3), atrial tachycardia (*n* = 3), ventricular tachycardia (*n* = 2) and supraventricular extrasystoles (*n* = 1) or junctional extrasystoles (*n* = 1) and led to non-indicated or delayed treatments in five patients. Both patients with the misdiagnosis of ventricular tachycardia had a history of coronary artery disease and ischemic cardiomyopathy with a persistent reduced systolic left ventricular function (LVEF 30% and 45%). These patients suffered from inadequate ICD shocks due to dual antegrade conduction or AVNRT. Misdiagnosis of AF resulted in subscription of oral anticoagulation and beta-receptor blocker therapy in two patients. Misdiagnosis of atrial tachycardia led to medical treatment with flecainide and propafenone as well as an electrophysiological study without obtaining the correct diagnosis in a further case. In this patient, correct diagnosis and treatment with ablation could finally be obtained in a second electrophysiological study, which was then performed in one of the participating centres of the present study.

In one patient, dual antegrade conduction was coincidentally observed during ablation of symptomatic drug-refractory AF, which was not directly treated due to absent clinical symptoms. Two years later the patient was still free of AF when a first supraventricular tachycardia occurred and was diagnosed as AVNRT, which was then treated successfully by slow pathway modulation.

**Fig. 3:**
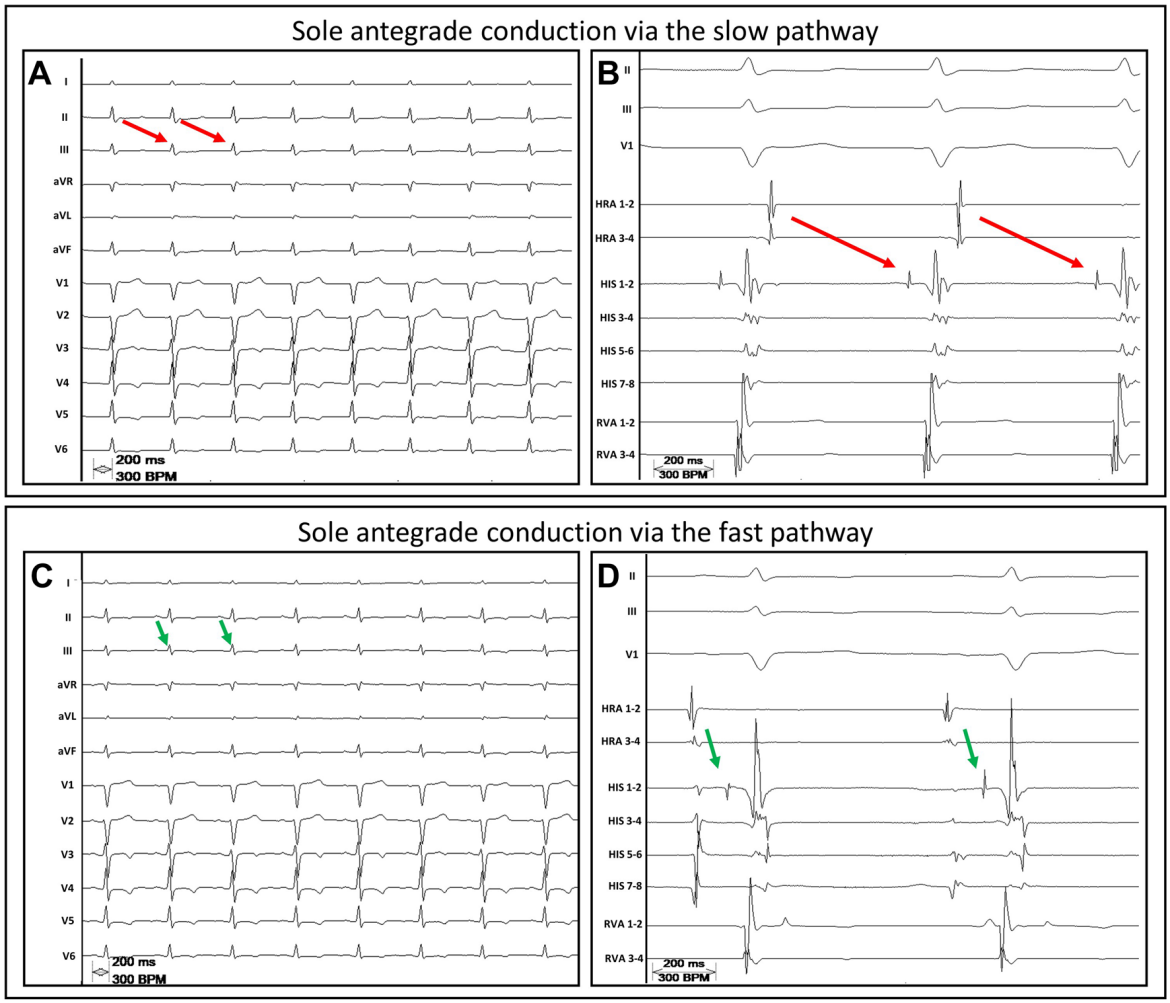
Modulation of the autonomic nervous system induces intermittent conduction via either the slow or the fast pathway or dual antegrade conduction. The red arrows indicate the slow pathway conduction. The green arrows indicate the fast pathway conduction. **a**, **c** A 12-lead ECG and **b**, **d** the corresponding intracardiac electrograms—from three bipolar catheters placed in the high right atrium (HRA), the right ventricular apex (RVA) and the His-bundle (HIS). **a/b** In a patient with DAVNNT and tachycardia-induced cardiomyopathy a period of sole antegrade conduction via the slow pathway is presented. **c/d** After intravenous administration of orciprenaline, sole fast-pathway conduction occurred. The latter was not induced by administration of atropine. Shortly afterwards a continuous change of dual antegrade conduction, sole slow-pathway and sole fast pathway conduction was observed. Note the great difference of the conduction times of the slow and fast pathway, which is thought to be a prerequisite in patients with dual antegrade conduction

One patient with initially reduced LVEF of 39%, which normalized after successful slow-pathway ablation, and no evidence indicating structural heart disease was diagnosed with a tachycardia-induced cardiomyopathy. During the electrophysiological study a recurrent change of conduction via solely the fast pathway, the slow pathway or dual ventricular response was observed and modulated by application of orciprenaline but not by atropine (Fig. 3).

In 12 out of 17 patients, an AVNRT (mean cycle length 378 ± 67 ms) could be induced by programmed stimulation with or without additive pharmacological provocation. Retrograde conduction could be demonstrated in six patients under baseline conditions or after administration of atropine or orciprenaline. Mean fast pathway conduction time was 138 ± 61 ms, and mean slow pathway conduction time was 593 ± 134 ms resulting in a mean conduction difference of 449 ± 113 ms (Table 2). In five patients with a diagnosis of DAVNNT during the electrophysiological study, an AVNRT was induced by programmed stimulation or occurred spontaneously.

**Table 2:** Electrophysiological parameters of all patients with dual antegrade conduction in the atrioventricular (AV) node

Patients #	Age (years)	Sex	Retrograde conduction	AH1 (ms)	AH2 (ms)	H1H2 (ms)	AVNRT
1	42	F	No	110	615	505	Yes
2	80	M	Yes	108	464	356	Yes
3	55	M	n.a	139	431	292	Yes
4	50	F	Yes	278	770	494	Yes
5	45	M	No	238	692	454	Yes
6	41	M	No	102	392	290	Yes
7	61	F	No	112	482	370	Yes
8	54	M	No	222	650	428	No
9	71	F	No	70	474	404	No
10	62	M	Yes	n.a	n.a	n.a	No
11	56	M	Yes	142	570	428	Yes
12	69	M	Yes	n.a	n.a	362	Yes
13	42	M	No	104	656	552	Yes
14	10	F	Yes	120	740	620	Yes
15	56	F	No	120	820	700	No
16	31	M	No	140	660	520	No
17	55	F	No	70	480	410	Yes
Mean ± SD	52 ± 16			138 ± 61	593 ± 134	449 ± 113	

*AVNRT* atrioventricular nodal re-entry tachycardia, *A* atrial activation, *F* female, *H1* first His signal, *H2* second His signal, *M* male

Despite the common initial misdiagnosis, the final correct diagnosis could be obtained by an electrophysiological study in all patients with concurrent safe and efficient slow-pathway modulation or ablation. All procedures were acutely successful and no major or minor adverse events occurred. Post-procedurally oral anticoagulation could be discontinued.

### Follow-up

During long-term follow-up of median 17 months (range 6–72 months, 18% with 24 h Holter monitoring) all patients reported symptom improvement. In one patient a recurrence of an enduring dual antegrade conduction occurred. In this patient, slow pathway ablation was primarily successful with short-term recurrence. Since about 2 months after the ablation, symptoms spontaneously disappeared and there have been no arrhythmias documented in the patient’s ICD for more than 5 years afterwards, so that no second electrophysiological study has been performed. In the remaining 16 of 17 patients no supraventricular tachycardia including AF was observed. One patient with a known dilated cardiomyopathy suffered from appropriate ICD shocks. Cerebrovascular events or AF were not observed in any of the patients during long-term follow-up.

## Discussion

To our knowledge, this is the first multicentre study in patients with dual antegrade conduction in the AV node. It covers about one-fifth of the so-far reported cases [8–12, 21–27]. We demonstrate that (1) catheter ablation in patients with dual antegrade conduction is safe, effective, and results in good patient outcome during long-term follow-up and that (2) a co-existence of dual antegrade conduction in the AV node and AVNRT is more common than previously thought. The latter appears to be related to changes in ante- vs. retrograde conduction via slow and/or fast pathway depending on autonomic tone, which can challenge the diagnosis and therapy in some patients.

In patients with supraventricular tachycardias related to dual antegrade conduction via the AV node, misdiagnoses are common (approx. 70%) and the average time to the final diagnosis is often longer than 1 year [8–10]. This may be explained by the challenging differential diagnosis, clinically asymptomatic patients or a lack of knowledge of its existence [9, 28]. Dual antegrade conduction can mimic a variety of other arrhythmias, e.g. AF, which is the most common misdiagnosis having repetitively resulted in non-indicated oral anticoagulation [8, 9]. Misdiagnosis as VT can also have severe consequences, e.g. inadequate ICD shocks by insufficient discrimination of the ICD systems [28–32]. Inadequate indication for ICD implantation might be even more important. Tachycardia-induced cardiomyopathy due to DAVNNT has been described as indication for ICD implantation for primary [33] and secondary prevention [34]. The final diagnosis can in most cases be relatively easy suspected by a 12-lead ECG and can finally be confirmed during an electrophysiological study. Due to the high number of misdiagnoses or asymptomatic patients, parameters as sustained, non-sustained or the arrhythmia burden have not been reported yet.

Considering a general underestimation combined with frequent misdiagnosis, mistreatment, and delay of correct diagnosis, we assume that this ‘chameleon of the AV node’ is of greater clinical relevance than previously thought. This is supported by a previous single-centre experience, in which we performed catheter ablation in 3 out of 231 patients with supraventricular tachycardia due to dual antegrade conduction via the AV node. Addressing dual antegrade conduction in future international guidelines in detail might be helpful to standardize diagnostic criteria and establish a universally accepted name like DAVNNT [2, 8, 9, 17, 18].

Until now, there have only been rare reports of a co-existence of dual antegrade conduction and AVNRT [8–10, 35, 36]. It has been hypothesized that some of the prerequisites for a DAVNNT, such as missing or poor VA conduction, missing or poor retrograde slow pathway conduction and/or a large difference in conduction velocities between the slow and fast pathway, make an AVNRT unlikely [9]. By contrast, we here observed a relatively high amount of patients with a co-existence of dual antegrade conduction and AVNRT (71% patients). This coincidence of dual antegrade conduction and AVNRT can be explained by the relatively high incidence of retrograde conduction as well as the great velocity difference of the slow and fast pathway. Besides this, the influence of the autonomic nervous system on the conduction system, which leads to different conduction properties at different times, may be causative [9, 36]. Depending on this initial evidence, one might speculate that the occurrence of this supraventricular tachycardia seems to depend on beta-adrenergic stimulation, as rapid changes in ante-/retrograde conduction as well as changes of conduction via the slow and fast pathways have been observed. This impact can be used as a diagnostic tool for the detection of dual antegrade conduction by pharmacological modulation of the autonomic nervous system via the stimulation of beta-adrenergic receptors or blockade of muscarinergic receptors [8].

Almost all patients with DAVNNT have been treated with slow pathway modulation or ablation [6, 8]. These case reports and two small single-centre experiences including three [14] and five patients [10], respectively, indicate high procedural success rates. Most of these patients were treated with radiofrequency ablation, only two patients underwent cryo-ablation [8, 9]. In one of the latter, a second procedure with radiofrequency ablation was necessary due to DAVNNT recurrence [8]. Overall, in only four patients a recurrence could be observed [8, 9, 22] and no major adverse events have been reported so far, especially no permanent AV block requiring a pacemaker implantation [8, 9, 22]. These numbers are similar to the reported success rate (> 95%) and adverse events (overall < 5%, risk for permanent AV block < 1%) of slow pathway ablation/modulation for AVNRT [4, 8]. Although successful long-term treatment with antiarrhythmic medication has been reported in one patient with DAVNNT, ablation therapy of the slow pathway should be considered as standard therapy in symptomatic patients [8, 9]. Co-incidence of dual antegrade conduction and other arrhythmias including AF is not well defined. Here, no AF or cerebrovascular events were observed in the long-term follow-up [37].

## Conclusion

This first multicentre study investigating patients with supraventricular tachycardia and dual antegrade conduction in the AV node demonstrates that catheter ablation is safe and effective while long-term patient outcome is good. It supports previous case reports demonstrating that this supraventricular tachycardia, induced by dual antegrade conduction in the AV node, mimics other more common arrhythmias and is often overlooked. Changes in ante- vs. retrograde conduction via slow and/or fast pathway depending on autonomic tone impact its clinical presentation and can challenge the diagnosis and therapy of this ‘chameleon of the AV node’, that will hopefully be addressed in detail in future international guidelines.
